# Proliferation of *Bifidobacterium L80* under different proportions of milk protein hydrolysate

**DOI:** 10.1186/s12934-021-01702-3

**Published:** 2021-11-18

**Authors:** Bing Wang, Yang Yang, Xin Bian, Hua-nan Guan, Lin-lin Liu, Xue-xia Li, Qing-qi Guo, Wojciech Piekoszewski, Feng-lian Chen, Na Wu, Zhan-qian Ma, Yan-guo Shi, Na Zhang

**Affiliations:** 1 Key Laboratory of Food Science and Engineering of Heilongjiang Province, College of Food Engineering, Harbin University of Commerce, No. 1, Xuehai Street, Songbei District, Harbin, 150028 Heilongjiang People’s Republic of China; 2 Forestry School, Northeast Forestry University, No. 26, Hexing Street, Xiangfang District, Harbin, 150040 People’s Republic of China; 3 Department of Analytical Chemistry, Faculty of Chemistry, Jagiellonian University, Kraków, Poland; 4 Far Eastern Federal University, School of Biomedicine, FEFU Campus, Russian Island, Vladivostok, Russian Federation

**Keywords:** Whey protein hydrolysate, Casein hydrolysate, *Bifidobacterium L80*, Proliferation, Organic acid

## Abstract

The intestinal microecological environment is critical to an infant's growth. For those infants consuming milk power, it is very important to improve the intestinal microecological environment to promote the healthy growth of infants. In this paper, Milk protein hydrolysate (MPH), consisting of different proportions of proteins and small molecule peptides (5:5, 4:6, 3:7, 2:8, 1:9) were added to infant formula powder (IFP). The effects of MFP-enriched IFP addition on proliferation and metabolism of *Bifidobacterium L80* were studied. Compared with MPH-free IFP, MFP-enriched IFP with 1:9 of proteins to small molecule peptides significantly enhanced the proliferation of *Bifidobacterium L80*, resulting in higher cell density, greater viable counts and higher titratable acidity. MFP-enriched IFP increased the content of seven organic acids and H_2_O_2_ in the system, and improved the antibacterial activity to *E. coli BL21*. This study suggested that MPH could be an effective addition to infant formula powder to promote the growth of *Bifidobacterium*, so to improve the intestinal health of infants.

## Introduction

Neonatal intestinal colonization, a critical stage for an infant’s future physiological health, is beneficially influenced by breastfeeding [38]. Breast milk contains all the nutrients and microbial flora that an infant needs. In cases of breastfeeding insufficient or impossible, cow mike is often used as an alternative. However, cow milk does not contain probiotics. It is of industrial significance to develop formula milk powder which is beneficial to the establishment and increment of infant intestinal flora.

*Bifidobacterium* is a critical constituent of the human microbiome and plays important a role in digestion and gut immunity, which can benefit the host by improving the balance of intestinal microbes and bring health benefits [6, 8]. Infant’s gut microbiota, which plays a critical role in early life [9], is rich in *Bifidobacterium* [26], when *Bifidobacteria* are deficient in the gut, stimulation of their proliferation is required. Recent researches have investigated the effects of oligosaccharides [41], polysaccharides [15], peptides [24, 46], unsaturated fatty acids [30], cyanidin rutinoside [17, 31] on the proliferation and metabolisation of *Bifidobacteria*.

Research has shown that ɑ-Lactalbumin [2], β-lactoglobulin [45], lactoferrin [28], κ-casein [43] and other proteins or peptides can promote *Bifidobacteria* growth [20]. In particular, it has been reported that MPH containing short peptides [33] could improve the *Bifidobacteria* content in foods containing probiotics. However, the effect of the mixture of peptides (as determined by the extent of hydrolysis of the protein precursors) on the proliferation and metabolism of *Bifidobacteria* is unknown.

*Bifidobacterium* has an important inhibitory effect on *Listeria monocytogenes, Clostridium, staphylococcus, Enterococcus faecalis* and *Bacillus* [1, 27, 35]. *Bifidobacterium* display a wide range of antimicrobial activities. Besides the production of lactic acid and acetic acid, it also produces fatty acids, hydrogen peroxide, diacetyl and other substances, which play antibacterial role [1, 29].

In conclusion, the increment and antibacterial activity of *Bifidobacterium* can promote the intestinal functional development of infants to some extent. The object of this research was to assess the influence of the MPH infant formula on the proliferation and metabolism of *Bifidobacterium L80*, and the inhibition of Escherichia *coli BL21*. To lay a foundation for the development of infant formula milk powder conducive to healthy intestinal construction.

## Materials and methods

### Reagents and materials

Whey protein was obtained from Zhengzhou Jiangda Biological Technology Co., Ltd (Zhengzhou, China). Casein was obtained from Beijing Aoboxing Biotechnology Co., Ltd., (Beijing, China). Alcalase® 2.4 L FG 208000 U/g, Sigma Chemical Co., St. Louis, MO, USA was preserved at − 4 °C. *Bifidobacterium L80* were provided by the Laboratory of Harbin University of Commerce. Commercially available IFP for comparison was purchased from Wandashan Dairy Co., Ltd.,(Harbin, China).

Analytical standard-grade acids were obtained from Germany (Dr. Ehrenstorfer Gmb-H). Stock standard solutions were prepared by dissolution of acids in Milli-Q® (generated by use of a Milli-Q® system from Millipore (Milford, MA, USA)); all solutions were stored at 4 °C and were used within 1 month. Working standard solutions were prepared daily by dilution with Milli-Q®. Metaphosphoric acid and methanol were chromatographic-reagent grade and obtained from Merck (Sartorius, Gettingen, Germany). Whatman cellulose membrane filters (0.45 µm) were obtained from Millipore Co., USA.

### Preparation of whey protein and casein hydrolysate

Milk protein hydrolysates (MPH) with different ratios of protein to peptide were prepared by adjusting the amount of added enzyme. The whey protein and casein powder were mixed into deionized water to make a solution of whey protein and casein at a concentration of 5% w/v. The temperature was set to 50 °C for the hydrolysis and the pH was adjusted to 8.5 by solution of 2 M NaOH. Alcalase® was added at varying ratios (1000U/mL, 1500U/mL, 2000U/mL, 2500U/mL, 3000U/mL) to the mixture of whey protein and casein. After 4 h, the solution was heated to 95 °C and kept for 10 min to kill enzyme, and then was cooled down to room temperature. Then the pH was readjusted to 7 with 2 M NaOH. The hydrolysates were centrifuged at 7000 r/min for 30 min. The resulting milk protein hydrolysate was under freeze drying and was cryopreserved for later usage as supplementation of the IFP. The infant formulation powder was prepared according to the “National Food Safety Standard for Infant Formula Food”. MPH was mixed with IFP at a ratio of 12% (w/w) to provide MPH-enriched IFP with different proportions of proteins and peptides (5:5, 4:6, 3:7, 2:8, 1:9 w/w).

### Analytical methods

#### Determination of growth of *Bifidobacterium L80*

MPH-enriched IFP with different proportion of protein and peptide (5:5, 4:6, 3:7, 2:8, 1:9 W/W) were added to MRS medium as the experimental group. MPH-free IFP were used as a blank group, the commercial IFP were used as a control group. The mixture was sterilized at 121℃ for 15 min. *Bifidobacterium L80* was inoculated into the experimental group, the blank group and the control group were placed in a 37℃incubator for cultivation for 24 h. The viable count was implemented by plate-counting method [5].

The turbidimetric method was used to measure the growth of *Bifidobacterium L80*. Every 4 h (0 h, 4 h, 8 h, 12 h, 16 h, 20 h, 24 h), the optical density at 600 nm (OD600) was measured for cell growth using a spectrophotometer (UV-1600PC) [40].

#### Determination of metabolites

##### Determination of titration acidity

Titration acidity was measured according to Trinder [40]. During the growth of the *Bifidobacterium L80*, the titration acidity of the fermentation broth was measured at 0 h, 4 h, 8 h, 12 h, 16 h, 20 h and 24 h respectively. Phenolphthalein was used as indicator. The *Bifidobacterium* fermentation broth was titrated with 0.1 M NaOH to reddish color, and the red color did not disappear within 30 s. Acidity value (°T) is calculated based on the consumption of NaOH solution.

$${\text{Acidity value }}\left( {^\circ {\text{T}}} \right) = {\text{V}}_{{{\text{NaOH}}}} \times {2}0$$

##### Determination of the inhibitory ratio of *Bifidobacterium L80* to *E. coli*

The inhibitory ratio of *Bifidobacterium L80* culture on *E. coli* was measured at 24 h culturing using the Oxford cup method [36]. *E. coli* was used as indicator bacteria. *Bifidobacterium L80* of the experimental group, blank group and control group were cultured for 48 h, and then injected into Oxford cup for 24 h at 37 ℃. The size of inhibition zones was measured.

##### Identification of organic acids by HPLC

The MPH group was used as sample group, MPH with 1:9 proportion of macromolecules to small molecule peptides was added to the basic MRS medium. The milk protein group was used as control group, milk protein was added to the basic MRS medium. The basic MRS medium was used as blank group. During the growth of the *Bifi*dobacter*ium L80* in different groups, Organic acid (Formic acid, Malic acid, Acetic acid, Tartaric acid, Lactic acid, Citric acid) metabolized by *Bifidobacterium L80* from the sample group, the blank group, the control group were measured by HPLC at 0, 12, 24, 36, 48, 60, and 72 h.

A HPLC system (Waters® 2695, USA), the parameters of the experiment were as follows: photodiode array detector (Waters® 2489), Agilent ZORBAX SB-C18 column (250 × 4.6 mm, 5 µm), Empower® Chromatography Workstation software. Meanwhile, isocratic elution program (0.1% v/v H_3_PO_4_ and methanol) with a flow rate of 1 mL/min was used in this test. Absorbance was monitored at 210 nm and the sample injection volume was 10 μL.

##### Determination of the hydrogen peroxide content

In acidic environment, hydrogen peroxide reacts to with titanium ions to form an orange complex. The amount of hydrogen peroxide in the sample is then determined by titanium salt spectrophotometry at 430 nm [23]. 10 mL samples and 5 mL titanium ions were transferred to a colorimetric tube, water was added to colorimetric tube to 25 mL. The solution was shaded well and then left still for 10 min. The blank group was replaced with water. The absorbance value was measured at 430 nm.

### Statistical analysis

Data were processed and plotted using Excel 2010. The results were analysed using SPSS 17.0 software. All experiments were conducted in triplicate. Analysis of variance (ANOVA) and minimum significant difference (LSD) were performed on the data (*P* < 0.05).

## Results and discussion

### Proliferation assessment of *Bifidobacterium L80* with proteins and small molecule peptides

**Fig. 1:**
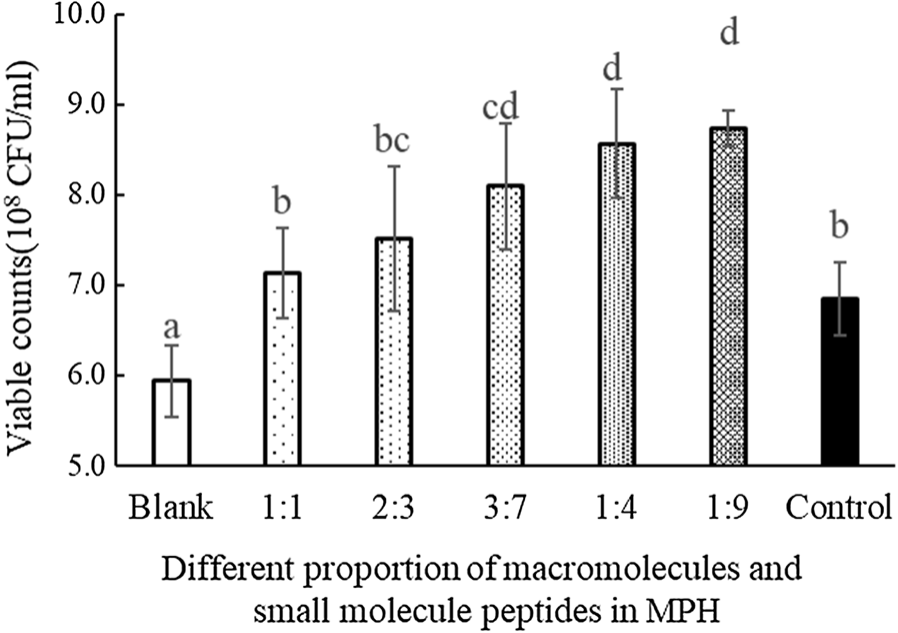
Effect of different proportion of macromolecules and small molecule peptides in MPH on viable counts after 24 h of incubation. In the figure, 1:1, 2:3, 3:7, 1:4, 1:9 refers to the ratio of macromolecules and small molecule peptides at MPH. ^a~d^ indicates a significant difference between viable counts at different proportion of macromolecules and small molecule peptides (P < 0.05)

The effects of different ratios of protein to small molecule peptide on the 600 nm optical density (OD600) and the viable count of *Bifidobacterium L80* are shown in Table 1 and Fig. 1, respectively.

**Table 1:** Change of optical density (OD) value in the control and mixtures of infant formula containing different proportion of macromolecules and small molecule peptides in MPH

Macromolecules/small molecule peptides	0 h	4 h	8 h	12 h	16 h	20 h	24 h
Blank	0.071 ± 0.019^Aa^	0.128 ± 0.070^Ba^	0.344 ± 0.080^Ca^	0.619 ± 0.086^Da^	0.687 ± 0.073^Ea^	0.696 ± 0.079^Ea^	0.700 ± 0.038^Ea^
5:5	0.073 ± 0.018^Aa^	0.128 ± 0.069^Ba^	0.349 ± 0.075^Cb^	0.748 ± 0.084^Db^	0.881 ± 0.121^Eb^	0.894 ± 0.745^Ea^	0.890 ± 0.062^Eb^
4:6	0.071 ± 0.010^Aa^	0.128 ± 0.047^Ba^	0.348 ± 0.047^Cb^	0.791 ± 0.062^Dc^	0.919 ± 0.067^Eb^	0.922 ± 0.841^Eb^	0.922 ± 0.130^Eb^
3:7	0.079 ± 0.013^Aa^	0.131 ± 0.074^Ba^	0.349 ± 0.082^Cb^	0.858 ± 0.084^Dd^	0.955 ± 0.105^Eb^	0.959 ± 0.110^Eb^	0.960 ± 0.129^Ebc^
2:8	0.071 ± 0.015^Aa^	0.133 ± 0.047^Ba^	0.343 ± 0.028^Ca^	0.873 ± 0.106^Dd^	0.990 ± 0.068^Ec^	0.979 ± 0.103^Ec^	0.999 ± 0.097^Ec^
1:9	0.070 ± 0.020^Aa^	0.145 ± 0.036^Bb^	0.356 ± 0.097^Cc^	0.951 ± 0.103^De^	1.073 ± 0.096^Ee^	1.073 ± 0.123^Ed^	1.079 ± 0.080^Ed^
Control	0.071 ± 0.010^Aa^	0.232 ± 0.083^Ba^	0.352 ± 0.027^Cb^	0.902 ± 0.133^Dd^	0.917 ± 0.100^Eb^	0.922 ± 0.099^Eab^	0.921 ± 0.037^Eb^

^A^^~^^E^ indicates a significant difference between OD value at different culture times (*P* < 0.05)

^a^^~b^ indicates a significant difference between OD value at different proportion of macromolecules and small molecule peptides in MPH (*P* < 0.05)

During 0–16 h, the OD600 of the culture solution increased significantly and then became stable (i.e., no significant proliferation of *Bifidobacterium L80*) between 16 to 24 h. At 24 h, the OD600 of the culture treated with IFP containing MPH at a 1:9 of proteins and small molecule peptides was 1.079 ± 0.080. As shown (Fig. 1), the viable count at 24 h in MPH-enriched IFP comprising a 1:9 proportion of proteins and peptides (8.74 × 10^8^ cfu/mL) was significantly greater than that in the blank group (5.94 × 10^8^ cfu/mL) and that in the control group (6.85 × 10^8^ cfu/mL) (*P* < 0.05). From the data in Table 1 and Fig. 1, it can be seen that the concentration of viable bacteria was related to the proportion of proteins and peptides in the MPH. MPH-enriched IFP with the protein to small molecule peptide ratio of 1:9 had the best proliferation on *bifidobacterium L80*. The OD600 of *bifidobacterium L80* was the highest (indicating the greatest proliferative effect; equating to 1.54 times and 1.17 times the value for the blank group and the control group, respectively). The viable counts in the blank group were 1.47 times and 1.28 times as many as those in the control group, respectively, which were not considered significantly different.

Li et al. [25] used soybean protein and soybean peptides digested in vitro to prepare different nitrogen-containing media for the culture of *Animal Bifidobacterium Subspecies*. The results showed that soybean protein and soybean peptide could promote the growth and metabolism of *Animal Bifidobacterium Subspecies*. The effect of digesting soybean peptides was considered better. Bai et al. [4] analysed soybean protein (MW < 5000 Da) hydrolysates that’s rich in short peptides for the presence of 20 different full-valent amino acids in their complement of free amino acids. Although the amount of soybean protein hydrolysate added to the cultures contained only one third of the 20 amino acids, the maximum growth rate of these cultures was fivefold greater than that of the cultures treated with all 20 full-valent amino acids. The small molecular weight is not the whole reason for promoting the proliferation of probiotics. The results showed that the greatest growth-rate-enhancing hydrolysate was that with the richest nitrogen source that could be most easily used by lactic acid bacteria. These bacteria have low protease activity, so it is necessary to provide cultures with essential amino acids, vitamins and other growth factors, depending on the growth environment, to obtain high-density cell cultures.

Other researchers have emphasised the role of oligopeptides: Tynkkynen et al. [39] suggested that 98% of the nitrogen source for *Lactococcus lactis* growth relies on oligopeptides in milk, in which oligopeptide transport system plays a key role. Pritchard et al*.* [32] proved that the proliferation of lactic acid bacteria relies on dipeptides and tripeptides, in addition to six amino acids, to form oligopeptide systems. Although all of these systems involve the absorption of essential amino acids in nutrient media, the highest absorption rate is achieved by transporting dipeptides and tripeptides. Therefore, it’s reasonable to infer that proteolysis products with the highest content of short peptides (dipeptides and tripeptides) have the greatest growth-enhancing effects on the growth of *Lactobacillu*s spp.

The role of lactoprotein also depends on the performance of *Lactobacillus* proteolytic enzymes and peptide transport systems, according to recent studies [7, 19, 44]. Lactobacillus growth in milk was found to depend on its protein hydrolysis system: casein was degraded by enzyme to form polypeptide fragments, oligopeptides are transported into cells by transporters, intracellular peptides was degraded into amino acids by enzyme. Kieliszek et al*.* [21] and Lazzi et al. [22] came to similar conclusions. Study of the proliferation experiments of lactic acid bacteria with functional feather proteins, showed that most of the soluble proteins of functional feather proteins are composed of smaller, soluble peptides. We need to further verify and analyse the various opinions about the reasons for the proliferation of probiotics promoted by lactin peptides.

The effect of MPH containing high levels of peptides on the titration acidity of *Bifidobacterium L80* medium is shown in Table 2. The acidity of the culture fluids in each group increased rapidly over 0–16 h, which may be due to rapid proliferation generating a high concentration of acidic metabolites. Over the 16–24 h period, the increase in acidity was not significant, which could indicate that during this period of time, nutrients required by *Bifidobacterium L80* (e.g., sugars) were almost exhausted, or the *Bifidobacterium L80* culture was in decline. Overall: in the beginning, rapid bacterial growth during the logarithmic phase (as indicated by the steep OD_600_ curve during this period) increased the acidity of the system, and then short peptides generated by proteolysis promoted bacterial growth and thus increased the acid production capacity of *Bifidobacterium L80*. In Table 2 it shows that that acidity stabilised at approximately 16 h, indicating that *Bifidobacterium L80* is at the end of its stable growth period. This slow growth can be attributed to the depletion of nutrient supplies, and the inhibition of growth by higher concentration of acidic metabolites.

**Table 2:** Effect of different proportion of macromolecules and small molecule peptides in MPH on titratable acidity during 24 h of incubation

Macromolecules/small molecule peptides	0 h	4 h	8 h	12 h	16 h	20 h	24 h
Blank	23.10 ± 1.09^Aa^	32.00 ± 1.09^Aa^	52.20 ± 0.95^Ca^	52.80 ± 1.09^Ba^	61.80 ± 1.19^Ea^	65.10 ± 1.78^Ea^	79.00 ± 2.44^Ea^
5:5	23.10 ± 2.14^Aa^	35.90 ± 1.09^Aa^	55.80 ± 2.53^Ca^	58.80 ± 1.09^Ba^	67.80 ± 1.32^Ea^	79.10 ± 2.36^Ea^	87.00 ± 2.66^Ea^
4:6	23.60 ± 3.13^Aa^	38.30 ± 1.09^Aa^	58.80 ± 1.61^Cb^	63.70 ± 1.09^Ba^	73.70 ± 2.23^Eb^	84.40 ± 2.06^Ea^	87.82 ± 1.63^Eb^
3:7	23.90 ± 2.37^Aa^	38.50 ± 1.09^Aa^	58.40 ± 3.05^Cb^	72.40 ± 1.09^Ba^	78.50 ± 2.06^Eb^	84.30 ± 2.06^Ea^	93.60 ± 1.76^Eb^
2:8	23.50 ± 3.99^Aa^	39.80 ± 1.09^Aa^	60.80 ± 2.91^Cb^	79.50 ± 1.09^Ba^	87.00 ± 1.75^Eb^	85.10 ± 1.85^Ea^	94.55 ± 2.35^Eb^
1:9	23.2 ± 2.47^Aa^	41.2 ± 1.09^Aa^	61.20 ± 2.76^Cb^	71.2 ± 1.09^Ba^	83.00 ± 2.95^Eb^	86.8 ± 2.06^Ea^	95.6 ± 6.26^Eb^
Control	23.70 ± 3.01^Aa^	38.50 ± 1.09^Aa^	51.20 ± 3.98^Cb^	58.00 ± 1.09^Ba^	63.70 ± 4.33^Eb^	68.60 ± 2.23^Ea^	71.90 ± 3.05^Eb^

^A^^~^^E^ indicates a significant difference between the titratable acidity at different culture times (*P* < 0.05)

^a^^~b^ indicates a significant difference between the titratable acidity at different TCA-NSI MPH (*P* < 0.05)

After incubation for 24 h, the titration acidity of the blank and control groups cultures increased to 79.0°T and 71.9°T, respectively, compared to an increase in cultures treated with MPH-enriched IFP of formula powder (containing a 90% proportion of proteins and small molecule peptides) of 95.6°T, which was significantly different (*P* < 0.05). From the growth and acidity change of *Bifidobacterium L80*, it can be observed that continuous production of acid also has a significant effect on the viability of *Bifidobacterium L80*; i.e. the concentration of bacteria decreased significantly after 24 h, indicating that the acid environment (lactic acid) is beneficial to probiotics, but only up to a certain point. Excessive acidity will have a bad effect on the activity of the bacteria, and the effect of acidity on viability is direct, and is consistent with the results reported by Dave and Shah et al*.* [10].

### Inhibitory of *Bifidobacterium L80* to *E. coli BL21*

**Fig. 2:**
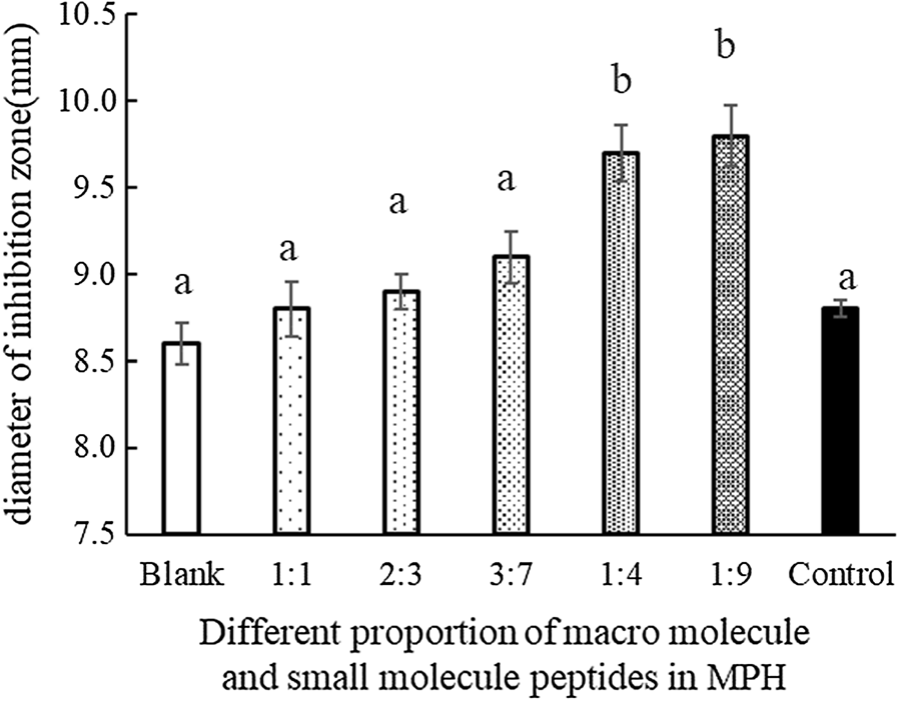
Effect of different proportion of macromolecules and small molecule peptides in MPH on *Bifidobacterium L80* and thus the effect of the metabolites on the inhibition of *E. coli*. In the figure, 1:1, 2:3, 3:7, 1:4, 1:9 refers to the ratio of macromolecules and small molecule peptides at MPH. ^a~b^ indicates a significant difference between the metabolites on the inhibition of *E. coli.* at different MPH addition (*P* < 0.05)

Different proportions of proteins and small peptides of MPH showed different inhibitory effects on *E. coli BL21*, as shown in Fig. 2. It was observed that an increase in the proportion of proteins and small peptides in the MPH-enriched IFP led to an increase in the diameter of the inhibition zone of *E. coli*, showing that an increased proportion of proteins and small molecule peptides in MPH promotes the antibacterial action of *Bifidobacterium L80* in the IFP. For example, when the MPH in the IFP comprised 90% proteins and small peptides, the inhibition zone diameter was 9.80 mm, which was 1.20 mm and 1.00 mm longer than in the blank group and the control group, respectively.

Some studies have suggested that the addition of polypeptides and amino acids to the culture medium is an effective way to increase the growth rate of lactic acid bacteria, and that whey protein hydrolysates have a positive effect on the formation of extracellular polysaccharides and the reduction of antigenicity [18, 48].

Casein hydrolysates also have a promoting effect on probiotic growth and show different degrees of inhibition of harmful bacteria. Zhi et al. [47] continuously fed cyclic guanosine monophosphate (CGMP), of mice to CGMP, and found that after 15 days, levels of *Lactobacillus*, *Enterobacteriaceae* and *Bifidobacterium* were significantly increased (*P* < 0.01), whilst levels of coliform bacteria were significantly reduced (*P* < 0.05).

Studies have shown that the protective effect of lactic acid bacteria on foods is mainly due to the production of some active metabolites, such as organic acids, which can inhibit the growth of spoilage and harmful microorganisms by lowering the pH of the system, as well as other substances such as ethanol, fatty acids, hydrogen peroxide, diacetyl, propionate, and phenyl lactic acid. Cyclic dipeptides and 3-hydroxy fatty acids, bacteriocins, and bacteriocins are also known playing a role in this protective effect [11, 37].

### Metabolite analysis

#### Evaluation of the organic acid content

**Fig. 3:**
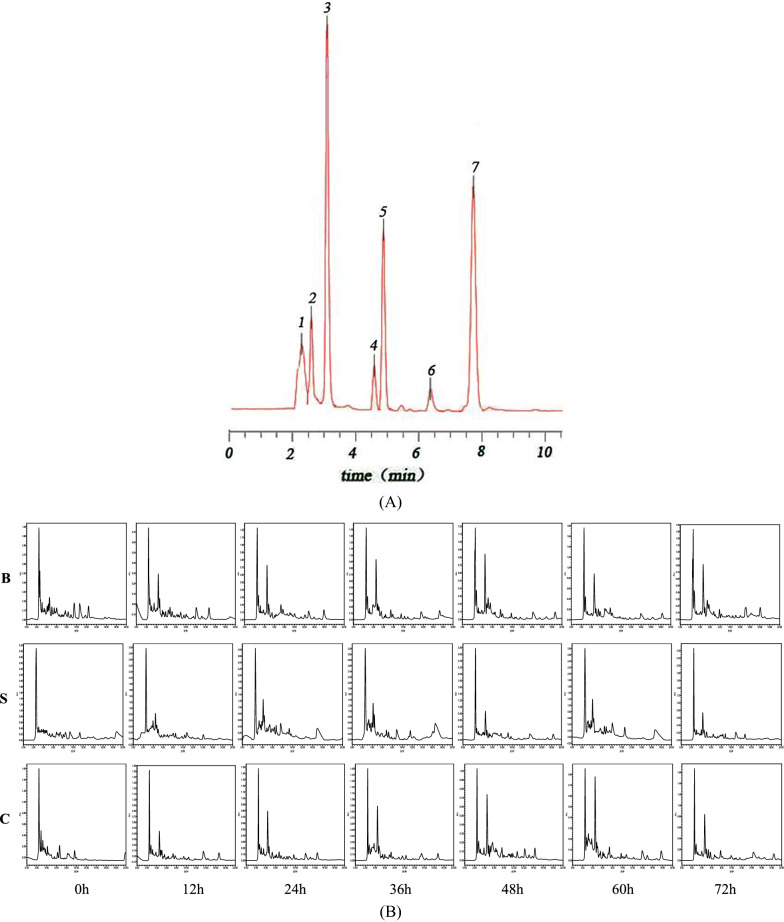
HPLC analysis of organic acid. **A** HPLC analysis of organic acid standard samples. Peaks: (1) formic acid; (2) oxalic acid; (3) malic acid; (4) acetic acid; (5) tartaric acid; (6) lactic acid; (7) citric acid. **B** HPLC analysis of organic acid produced by *Bifidobacterium L80* during 72 h in different medium. B: blank group; S: sample group; C:control group

HPLC chromatograms of seven organic acids are shown in Fig. 3, and the linear equation for the determination of organic acids is shown in Table 3.

**Table 3:** Linear correlation of organic acid determination

Peak number	Organic acid	Peak time(min)	Equation of linear regression	*r*^2^
1	Formic acid	2.403	y = 80640x + 3.210	0.981
2	Oxalic acid	2.745	y = 3543.7x + 14.869	0.988
3	Malic acid	2.957	y = 2199.22x + 0.9188	0.992
4	Acetic acid	3.594	y = 139.02x + 0.7112	0.996
5	Tartaric acid	3.957	y = 467.63x–1.565	0.973
6	Lactic acid	4.567	y = 1750.12x + 0.8043	0.999
7	Citric acid	6.309	y = 1884.03x-0.1525	0.987

The acid production of *Bifidobacterium L80* is observed by titratable acidity assay, and HPLC can accurately quantitate the organic acids produced by metabolism. The results are shown in Table 4 and Fig. 3.

**Table 4:** Changes of organic acids content between blank group, sample group and control group

Group	0 h	12 h	24 h	36 h	48 h	60 h	72 h
Formic acid							
S	375.23 ± 13.96^Aa^	346.60 ± 41.73^Aa^	363.71 ± 30.42^Aa^	316.24 ± 46.37^Aa^	296.80 ± 38.54^Ac^	368.52 ± 64.11^Aa^	323.46 ± 29.25^Aa^
C	132.55 ± 14.98^Ab^	136.46 ± 16.92^Aa^	148.86 ± 17.71^Aa^	171.44 ± 17.08^Ba^	172.66 ± 16.84^Ba^	180.43 ± 19.77^Ba^	170.89 ± 26.31^Ba^
B	161.66 ± 21.25^Aa^	169.25 ± 19.69^Aa^	162.05 ± 7.05^Aa^	175.36 ± 17.74^Ba^	179.44 ± 14.49^Ba^	176.04 ± 18.83^Ba^	182.36 ± 24.02^Ba^
Malic acid							
S	1509.57 ± 13.26^Aa^	1796.97 ± 47.06^Ba^	1713.53 ± 38.01^Ba^	1746.78 ± 26.62^Ba^	1795.79 ± 26.36^Ba^	3509.39 ± 6.55^Ca^	3964.10 ± 32.18^Ca^
C	984.97 ± 51.64^Ab^	1545.00 ± 4.17^Bb^	8824.06 ± 40.23^Cb^	4276.99 ± 73.35^Db^	3798.85 ± 10.08^Eb^	829.83 ± 87.97^Ab^	816.47 ± 36.04^Ab^
B	740.72 ± 19.22^Ac^	884.60 ± 18.61^Bc^	875.05 ± 39.19^Bc^	1107.02 ± 70.78^Cc^	1121.89 ± 46.17^Cc^	1203.91 ± 27.87^Cc^	1211.81 ± 33.16^Cc^
Acetic acid							
S	11,808.40 ± 37.46^Aa^	10,749.70 ± 71.55^Aa^	15,281.85 ± 36.09^Ba^	25,955.56 ± 24.28^Ca^	19,537.92 ± 46.78^ Da^	26,054.04 ± 46.61^Ea^	28,959.24 ± 53.51^Fa^
C	11,999.17 ± 48.58^Aa^	39,144.76 ± 54.20^Bb^	28,442.37 ± 72.75^Cb^	19,331.98 ± 30.87^Db^	37,808.13 ± 40.42^Ea^	13,416.85 ± 57.67^Fb^	14,136.17 ± 67.28^Fb^
B	11,979.74 ± 259.99^Aa^	11,712.70 ± 152.85^Ba^	22,185.18 ± 40.65^Cc^	26,274.13 ± 52.95^Dc^	26,223.01 ± 31.54^Ec^	23,345.78 ± 68.56^Fc^	20,390.12 ± 58.00^Gc^
Tartaric acid							
S	7076.60 ± 45.05^Aa^	10,936.73 ± 175.16^Ba^	8280.10 ± 129.98^Ca^	14,642.50 ± 162.01^ Da^	15,503.45 ± 190.70^Ca^	22,189.72 ± 17.13^ Da^	8028.57 ± 250.80^Ca^
C	3021.09 ± 69.46^Aa^	4423.68 ± 287.38^Bb^	2279.29 ± 146.66^Bb^	3634.52 ± 63.66^Bb^	11,095.15 ± 76.35^Bb^	6527.85 ± 22.87^Bb^	10,804.73 ± 78.58^Bb^
B	8080.52 ± 664.09^Aa^	4796.85 ± 698.35^Bb^	26,237.91 ± 279.37^Bc^	29,573.06 ± 453.82^Cc^	5593.38 ± 48.66^Dc^	33,504.45 ± 34.46^Ec^	4656.49 ± 24.51^Bc^
Lactic acid							
S	15,928.88 ± 38.46^Aa^	23,462.22 ± 98.99^Ba^	38,533.43 ± 31.55^ Da^	44,123.76 ± 216.39^Ea^	52,962.41 ± 64.50^Fa^	60,765.36 ± 73.53^ Ga^	683,687.47 ± 36.87^Ha^
C	19,988.31 ± 316.88^Ab^	13,108.94 ± 1240.51^Bb^	12,969.60 ± 257.78^Cb^	13,411.65 ± 1328.58^Bb^	12,128.05 ± 702.84^Bb^	11,317.57 ± 62.91^Bb^	11,190.02 ± 60.49^Bb^
B	13,539.66 ± 56.66^Aa^	14,016.28 ± 31.51^Bc^	14,262.22 ± 56.59^Cc^	48,697.84 ± 67.83^Dc^	59,608.36 ± 1291.34^Dc^	61,204.75 ± 1128.17^ Da^	63,937.61 ± 5136.01^Dc^
Citric acid							
S	2833.59 ± 250.87^Aa^	1827.42 ± 142.72^Aa^	507.97 ± 50.07^Aa^	539.52 ± 43.98^Aa^	188.95 ± 77.93^Aa^	1955.97 ± 47.73^Aa^	478.22 ± 28.26^Aa^
C	1301.16 ± 83.21^Ab^	1791.26 ± 50.82^Ba^	822.82 ± 40.27^Cb^	635.84 ± 186.69^Db^	849.45 ± 20.53^Cb^	1406.05 ± 62.47^Eb^	1545.00 ± 46.72^Eb^
B	748.19 ± 39.63^Ac^	613.27 ± 163.18^Ac^	430.99 ± 79.94^Bc^	1227.71 ± 46.07^Cc^	2060.72 ± 57.59^Dc^	797.49 ± 48.12^Ec^	549.27 ± 105.85^Fc^

In the figure, S refers to the sample group with 1:9 proportion of macromolecules and small molecule peptides in MPH addition to MRS medium; C refers to the control group with the milk protein addition to MRS medium; B refers to the blank group with the basic MRS medium. ^A−H^ Different alphabets indicate significant differences between different time at the same group (*P* < 0.05). ^a−c^ Different alphabets indicate significant differences between sample groups, control group and bland group within a row (*P* < 0.05)

The data in Table 4 show that concentration of acetic acid and lactic acid were the highest in the metabolites of *Bifidobacterium* cultures treated with MPH-enriched IFP after 72 h fermentation, being 28959.24 ± 53.51 and 683687.47 ± 36.87 mg/L, respectively. Additionally, concentrations of formic acid, acetic acid, malic acid, and lactic acid were also significantly higher than those of the blank and the control at that time. These results show that MPH-enriched IFP promotes probiotics to proliferate and generates primarily acetic acid (C2 chain-length) and lactic acid (C3 chain length) as metabolites, and that these short-chain organic acids have a positive antibacterial effect. Indeed, Rios-Covian et al*.* [34] concluded that Acetic acid was the most abundant organic acid formed followed by lactic acid, with moderate differences in production among strains; pyruvic, succinic and formic acids were also produced at considerably lower proportions, with variability among strains.

Blandino et al*.* [3] have verified by in vitro experiments that *Bifidobacteria* can produce organic acids and inhibit Gram-negative pathogenic bacteria.

#### Evaluation of the hydrogen peroxide content

**Fig. 4:**
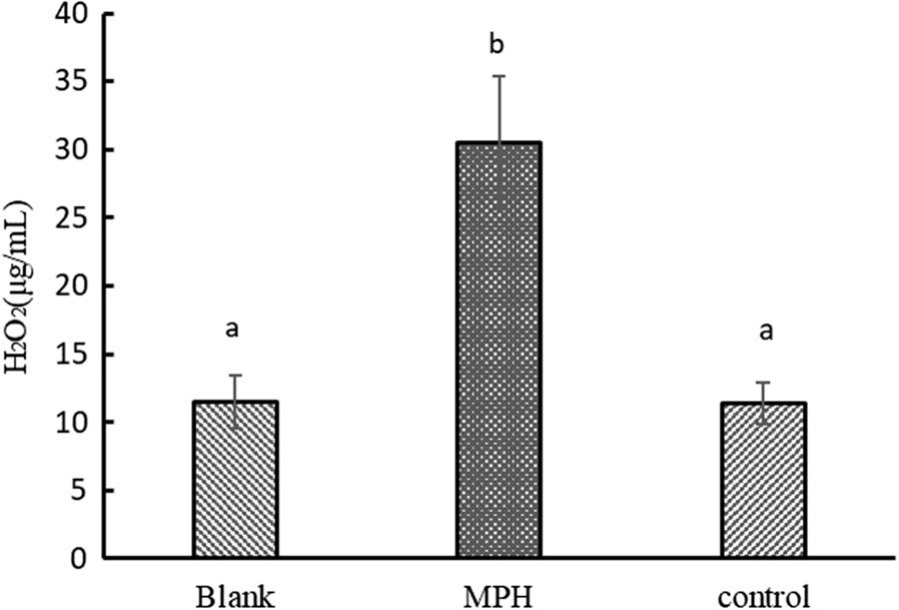
The content of H_2_O_2_ in the metabolites of *Bifidobacterium L80* fermentation broth of with 1:9 proportion of macromolecules and small molecule peptides in MPH cultured for 24 h. In the figure, MPH refers to the sample group with 1:9 proportion of macromolecules and small molecule peptides in MPH addition to MRS medium; Control refers to the control group with the milk protein addition to MRS medium; Blank refers to the blank group with the basic MRS medium. ^a−b^ Different alphabets indicate significant differences between sample group, control group and blank group within a row (P < 0.05)

The viability of probiotics is affected by pH value and also by hydrogen peroxide and dissolved oxygen [10, 14, 42]. The results of measuring the hydrogen peroxide (H_2_O_2_) content in the metabolite profile of the *Bifidobacterium L80* fermentation broth supplemented with MPH for 24 h are shown in Fig. 4.

The concentration of H_2_O_2_ in the fermentation broth of the MPH group is 30.49 µg/mL, significantly higher than those in the blank group and control groups. Under these conditions, the death of bacterial cells can be ascribed to the accumulation of H_2_O_2_-mediated irreversible oxidative damage of cell membranes, proteins, enzymes and DNA [12, 13]. These observations indicate that certain concentrations of extracellular H_2_O_2_ may serve bacteriostatic functions in host-defence mechanisms. Thus, this study confirms and extends the observations of Hiromi Kameya et al. [16], which concluded that H_2_O_2_ has bacteriostatic effects on *E. coli BL21*.

## Conclusions

All in all, MPH, as a supplement for IFP, has positive effect on the growth of *Bifidobacterium L80*. As the proportion of proteins to peptides in MPH increased, both cell density and viable counts of *Bifidobacterium L80*improved significantly. MPH also improved the inhibition of the growth of *E. coli BL21* by producing organic acids and H_2_O_2_. With proper selection of MPH additions, IFP has potential to afford nutritional and health benefits to infant. This study expanded the application of MPH-enrich IFP in the field of *Bifidobacterium*, provided a reference to the production of infant formula to improve infant’s intestinal health.

## Data Availability

The datasets used and analysed during the current study are available from the corresponding author on reasonable request.

## Abbreviations

MPH: Milk protein hydrolysate
IFP: Infant formula powder
CGMP: Cyclic guano sine monophosphate
